# Corrigendum

**DOI:** 10.1002/rcr2.166

**Published:** 2016-05-05

We would like to draw the reader's attention to an error in the following article:

Sekimoto Y , Kato M , Shukuya T , Koyama R , Nagaoka T , and Takahashi K . Bevacizumab‐induced chronic interstitial pneumonia during maintenance therapy in non‐small cell lung cancer. Respirol Case Rep. 2016; 4: e000151.10.1002/rcr2.151PMC481858327081491

The third author's first name was misspelled in the list of authors. The author's name should be listed as:

Takehito Shukuya

The publisher apologises for this error and any confusion it may have caused.

